# Arsenicum iodatum and arseicum album induces apoptosis and autophagy in glioblastoma multiforme

**DOI:** 10.6026/973206300214236

**Published:** 2025-11-15

**Authors:** Ankit Pateriya, Pratima Gupta, Ashutosh Shrivastava, Dhruv Varshney, Sreyanko Sadhukhan, Manendra Singh Tomar, Arun Kumar Gupta, Naibedya Chattopadhyay, Rohit Anthony Sinha

**Affiliations:** 1 Center for Advance Research, Faculty of Medicine, King George's Medical University, Lucknow-226003, Uttar Pradesh, India; 2 Department of Endocrinology, Sanjay Gandhi Postgraduate Institute of Medical Sciences, Lucknow-226014, Uttar Pradesh, India; 3 Division of Endocrinology and Centre for Research in Anabolic Skeletal Targets in Health and Illness (ASTHI), CSIR-Central Drug Research Institute, Lucknow-226031, Uttar Pradesh, India; 4 Academy of Scientific and Innovative Research (AcSIR), Ghaziabad- 201002, Uttar Pradesh, India; 5 Homeopathic Drug Research Institute, Lucknow-226010, Uttar Pradesh, India

**Keywords:** Glioblastoma multiforme, apoptosis, autophagy, homeopathic drugs

## Abstract

Glioblastoma multiforme (GBM) is a highly aggressive and treatment-resistant brain tumor, highlighting the need for novel therapeutic strategies. Therefore, it is of interest to investigate the anti-cancer effects of Arsenicum iodatum and Arsenicum album in U87 glioblastoma cells at 6C, 12C and 30C potencies. Both preparations induced apoptosis, confirmed by TUNEL along with the caspase-3 assays and triggered autophagy, evidenced by reduced p62 levels. Inhibition of autophagy enhanced cytotoxicity, indicating its protective role in GBM cells. Thus, we show that Arsenicum iodatum and Arsenicum album may serve as complementary candidates for GBM therapy, warranting further *in vivo* and mechanistic studies.

## Background:

Glioblastoma multiforme (GBM) is the most malignant and lethal form of brain tumor, with a median survival time of only 14.6 months and a dismal 5-year survival rate of less than 10% despite advances in neurosurgery, radiotherapy and chemotherapy [1, 2]. GBM arises from glial cells-the supportive cells of the central nervous system (CNS) that surround and protect neurons [3]. WHO recognizes two histopathological variants: large cell glioblastoma and gliosarcoma [4]. Although GBM affects fewer than 10 per 100,000 individuals worldwide, it accounts for nearly 80% of all malignant primary brain tumors, making it the most common and aggressive CNS malignancy [5, 6]. The prognosis of GBM remains extremely poor due to its highly infiltrative and invasive nature, which prevents complete surgical resection [7]. Radiotherapy, while standard, causes collateral toxicity to healthy brain regions, while chemotherapy is hampered by the blood-brain barrier (BBB) and drug resistance mechanisms of glioma cells [8, 9]. These barriers contribute to frequent therapeutic failure, highlighting the urgent need for alternative or complementary therapeutic strategies [10]. Arsenic has a long history in medicine, with documented therapeutic use for over 2400 years [11]. In modern oncology, arsenic trioxide (As_2_O_3_), derived from traditional Chinese medicine, has emerged as a clinically significant anti-cancer drug [12]. Its greatest success has been in acute promyelocytic leukemia (APL), where it induces remission by promoting apoptosis and differentiation of malignant promyelocytes [13, 14]. Preclinical studies also show that arsenic trioxide has broad-spectrum anti-tumor effects against hematologic malignancies and solid tumors, including those of the esophageal, gastric, lung and liver cancer [15]. These effects are mediated through reactive oxygen species (ROS) generation, mitochondrial dysfunction, metabolic interference and activation of programmed cell death pathways such as apoptosis and autophagy [16, 17- 18]. Homeopathy, a centuries-old therapeutic system using highly diluted substances, includes Arsenicum iodatum (derived from arsenic and iodine) and Arsenicum album (derived from arsenic trioxide), noted for their potential anticancer effects [19]. Although derived from toxic origins, potentization renders these preparations safe and while systematic research is limited, preliminary evidence suggests they may affect malignant cells through oxidative stress and apoptosis, yet no studies have explored their potential in GBM despite their chemical similarity to arsenic trioxide, an established anticancer drug [20, 21- 22]. Programmed cell death (PCD) remains a key therapeutic target in cancer. Apoptosis, characterized by caspase activation, DNA fragmentation and membrane blebbing, is exploited by many chemotherapeutic agents [23]. Autophagy, a cellular recycling process, has a dual role-supporting survival under stress but inducing death when excessively activated [24]. In GBM, both pathways are dysregulated, contributing to therapy resistance [25]. Therefore, it is of interest to report the anticancer potential of homeopathic arsenic-based preparations in GBM and investigating their ability to induce apoptosis and autophagy may offer a cost-effective adjunct to current therapies.

## Methodology:

## Cell Culture:

Human GBM U-87 MG Cells were cultured in α-Minimum Essential Medium (α-MEM; Gibco, USA) supplemented with 5% fetal bovine serum (FBS; Gibco, USA), 4 mM glutamine (Gibco, USA), 100 U/mL penicillin and 100 µg/mL streptomycin (Gibco, USA). Cultures were maintained at 37 °C in a humidified atmosphere containing 5% CO_2_. The absence of mycoplasma contamination was confirmed using a Mycoplasma Detection Kit (Takara Bio, Japan; RR277A).

## TdT-mediated dUTP nick end labeling (TUNEL) Assay:

Cells were seeded at a density of 2 x 10^4^ cells per well in 8-well chamber slides containing 300 µL of medium. After a 24-hour incubation to allow cell attachment, cultures were treated with the drug formulations (6C, 12C and 30C; 15 µL from stock) for 72 hours. Ethanol was used as the vehicle control. Apoptotic cell death was assessed using the TUNEL assay kit (Abcam, ab206386, UK). In this assay, terminal deoxynucleotidyl transferase (TdT) incorporates labeled nucleotides into DNA strand breaks, which are then visualized using the HRP-DAB detection system. Apoptotic cells were identified by the presence of brown nuclear staining [26].

## Caspase-3 colorimetric assay: 

Caspase-3 activity was measured using the Caspase-3 Colorimetric Assay Kit (Abcam, ab39401) following the manufacturer's protocol. Briefly, U87 cells treated with Arsenicum album or Arsenicum iodatum (6C) were harvested and lysed on ice for 10 minutes. Lysates were centrifuged at 10,000 x g for 10 minutes and 150 µg of protein from the supernatant was incubated with DEVD-pNA substrate in reaction buffer containing 10 mM dithiothreitol (DTT) at 37 °C for 2 hours. Caspase-3 activity was quantified by measuring p-nitroaniline release at 405 nm.

## Western blotting:

Cells were seeded at 2 x 10^5^ cells per well in 6-well plates and allowed to adhere for 24 h. After treatment with Arsenicum album or Arsenicum iodatum (6C); (5 µL from stock) for 24, 48 and 72 h, total protein was extracted. Equal amounts of protein (30 µg) were separated on 10% SDS-PAGE, transferred to PVDF membranes and probed overnight at 4°C with anti-p62 antibodies. After incubation with HRP-conjugated secondary antibodies, bands were detected using enhanced chemiluminescence, with GAPDH as a loading control [27].

## Cell viability assay:

A total of 3 x 10^3^ cells were seeded in 96-well plates and treated with either alone or in combination with a 50 µmol caspase inhibitor or 10nmol BafA1 for 1 hour and then incubated for 24 hours with Arsenicum iodatum or Arsenicum album (6C potency). Ethanol was used as the vehicle control. After 48 hours of treatment, MTT solution was added and incubated for 4 hours. The resulting formazan crystals were dissolved in DMSO and absorbance was measured at 570 nm [28].

## Results and Discussion:

A key feature of effective anticancer agents is their ability to induce PCD in malignant cells [29]. To assess this, we performed a TUNEL assay in GBM cells treated with Arsenicum album and Arsenicum iodatum. Both treatments caused a dose-dependent increase in TUNEL-positive cells, indicating DNA fragmentation. Arsenicum album-treated cells showed significantly higher apoptosis compared with controls (Figure 1A - see PDF) and similar DNA fragmentation was observed in Arsenicum iodatum-treated cells (Figure 1B - see PDF), demonstrating that these formulations induce apoptosis in GBM cells. Caspase-3, a key executioner of apoptosis, mediates DNA fragmentation and membrane blebbing [30]. To determine whether Arsenicum album induced apoptosis involves caspase activation, we performed a caspase-3 colorimetric assay. Arsenicum album treatment of GBM cells for 24 and 48 h caused a dose-dependent increase in caspase-3 activity, consistent with TUNEL results, confirming a caspase-dependent apoptotic mechanism. Similarly, Arsenicum iodatum also significantly elevated caspase-3 activity, indicating activation of the apoptotic pathway in GBM cells (Figure 2 - see PDF).

Autophagy often accompanies apoptosis in cancer cells exposed to cytotoxic agents, regulating cell fate [31]. The autophagy receptor p62 sequesters ubiquitinated proteins for degradation and its reduction reflects active autophagy [32]. In GBM cells, Arsenicum album and Arsenicum iodatum caused a time-dependent decrease in p62, confirming autophagy induction after 24 h (Figure 3A - see PDF) and after 48 h (Figure 3B - see PDF). While autophagy can initially promote survival, excessive activation contributes to cell death, enhancing the cytotoxic effect of Arsenicum iodatum. Next, we explored the crosstalk between apoptosis and autophagy in Arsenic-based drug-induced cytotoxicity (Figure 4A - see PDF and Figure 4B - see PDF). U87 cells were pretreated with 50 µmol/L caspase inhibitor for 1 hour and then incubated for 24 hours with Arsenicum iodatum or Arsenicum album (6C potency). Pretreatment with the caspase inhibitor significantly reduced cell death compared with drug treatment alone, indicating a caspase-dependent component. To further assess autophagy, we used 10 nmol BafA1, an inhibitor of vacuolar H^+^-ATPase, which blocks autophagosome-lysosome fusion [33]. Cell viability assays showed that BafA1 markedly increased Arsenicum iodatum- and Arsenicum album-induced cytotoxicity, suggesting that autophagy inhibition enhances cell death. These findings demonstrate that autophagy exerts a cytoprotective role in U87 glioblastoma cells and its inhibition sensitizes cells to Arsenicum-induced apoptosis. This study provides the first evidence that Arsenicum iodatum and Arsenicum album, homeopathic arsenic-based preparations, exert potent cytotoxic effects against U-87 GBM cells by activating PCD pathways. Mechanistically, Arsenicum Iodatum induced caspase-dependent apoptosis, demonstrated by dose-dependent increases in TUNEL-positive nuclei and enhanced caspase-3 activity, consistent with previous reports on arsenic compounds. Both treatments also decreasedp62 expression, indicating autophagy induction. Notably, autophagy inhibition with BafA1 enhanced Arsenicum Iodatum and album-induced cytotoxicity, suggesting that autophagy acts as a cytoprotective mechanism in GBM cells. Collectively, these findings reveal that arsenic-based homeopathic preparations modulate both apoptosis and autophagy, with autophagy inhibition tipping the balance toward enhanced apoptotic death. Given GBM's resistance to conventional therapies, these agents hold potential as complementary treatments. Further *in vivo* studies and mechanistic analyses are warranted to validate their translational relevance.

## Funding statement:

This work is funded by Central Council for Research in Homeopathy-Ministry of AYUSH, Government of India (Project No. S-14015/6/2019/SCHEME).

## Any conflict of interest:

Authors declare no conflict of interest.

## Abbreviations:

BafA1 - Bafilomycin A1

BBB - Blood Brain Barrier

CI - Caspase Inhibitor

CNS - Central Nervous System

GBM - Glioblastoma Multiforme

TUNEL - TdT-mediated dUTP nick end labeling

WHO - World Health Organization
